# Grief intensity following adolescent miscarriage or abortion: A descriptive study of recollections of adult women

**DOI:** 10.1016/j.heliyon.2024.e33634

**Published:** 2024-07-02

**Authors:** Caroline A. Lloyd, Marianne H. Hutti

**Affiliations:** a School of Education, Trinity College Dublin, Ireland; b College of Nursing, University of Kentucky, KY, USA; c Ambulatory Services, University of Kentucky (UK) Healthcare, KY, USA; d School of Nursing, University of Louisville, KY, USA

**Keywords:** Abortion, Adolescent, Grief, Miscarriage, Perinatal, Pregnancy, Descriptive research

## Abstract

**Objective:**

The purpose of this study was to examine women's recollected lifespan perceptions of the effect of grief intensity following adolescent perinatal death.

**Participants:**

Nineteen adult women who had experienced either a miscarriage or an abortion during adolescence. The study involved the recollection of events surrounding the experience which had occurred between three and 28 years previously.

**Methods:**

A 55-item online survey was used to gather recollected perceptions of adolescent miscarriage and abortion experiences. The Perinatal Grief Intensity Scale was embedded within this survey. The participants were instructed to recall their responses to the perinatal loss at the time of the event as an adolescent (T1; Time 1) and how they feel currently as adults about their previous adolescent perinatal death (T2; Time 2). Data were collected at both T1 and T2. The Perinatal Grief Intensity Scale is accompanied by an appropriately weighted Excel scoring sheet which was utilised to analyse the data at both T1 and T2.

**Results:**

As adolescents, participants perceived similar high and medium grief intensity when compared by type of loss (miscarriage, n = 6; abortion, n = 6). However, more women who had an abortion (n = 5) experienced low grief intensity compared with participants who had a miscarriage (n = 2). As adults, participants continued to perceive similar high and medium grief intensity when compared by type of loss (miscarriage, n = 6; abortion, n = 5). In addition, women who had an abortion continued to experience more low grief intensity (n = 6) compared with participants who had a miscarriage (n = 2). Approximately one quarter of adult female respondents, 26 % (n = 5) exhibited increased grief intensity as measured by the scores over time. Thirty seven percent (n = 7) exhibited no change in scores, and 37 % (n = 7) exhibited decreased scores over time in response to adolescent miscarriage or abortion.

**Conclusions:**

Support for the adoption of the Perinatal Grief Intensity Scale to identify women in need of follow-up for grief intensity after an adolescent miscarried or terminated pregnancy is evident. The results of this study have demonstrated that grief can resurge or appear in adult females as they respond to events across the lifespan, including further reproductive experiences. Therefore there is a compulsion for health care professionals to identify women at risk of intensive grief responses due to previous contributory events.

**Tweetable abstract:**

Healthcare providers should screen adult women who have experienced a previous adolescent miscarriage or termination for adverse mental health issues in adulthood.

## What is already known

• Estimates from The World Health Organization suggest that globally, approximately nine million adolescent girls over the age of 15 experience a perinatal death every year.
• There is an abundance of literature focused on adult perinatal loss responses, however, a literature review revealed there is a significant lack of information on perinatal grief during adolescence, and no literature was found that examined the long-term adult impact of a previous adolescent perinatal loss.

## What this paper adds

• This study has found that, for some women, the adverse psycho-social consequences of adolescent perinatal loss are enduring and grief may increase in response to adult reproductive events.
• This study found that women want to self-identify (or not) as a “bereaved parent”, suggesting that universal assumptive conference of “parent” identity is not appropriate for all women who experience an adolescent perinatal death.
• The findings from this study emphasise the need for healthcare professionals to identify adult women who have experienced an adolescent miscarriage or termination, to screen for adverse mental health issues in subsequent pregnancies.

## 1 Background

The objective of this study was to examine adult women's recollected level of grief intensity following a perinatal death loss, previously experienced as adolescents, with an eminent instrument. An additional objective was to compare women's recollections of their adolescent perinatal loss, to their current perception of the miscarriage or abortion as adults, utilising the same instrument, to identify any changes in grief response.

The current study defines perinatal loss as including early fetal death via miscarriage or elective abortion, late fetal death/stillbirth, and the post-pregnancy loss of neonatal death. The term “perinatal” is oft referred to, but ill defined. Whilst definitions encompass miscarriage, stillbirth, and neonatal death, the term is ambiguous. For example, one definition presented by Barfield (2011) [1] encompasses gestational ages between 20 weeks in utero until 28 days’ post birth, but the World Health Organization defines “perinatal” from the gestational age of 22 weeks until 7 days post birth [2]. Whilst the emotional responses to biological deaths (i.e. miscarriage, stillbirth, and neonatal deaths) have been widely researched and acknowledged in Euro Western and colonial cultures, empirical data investigating grief responses to abortion is sparse [3]. Within countries such as Japan, Thailand, and China all deaths from conception through to the neonatal period are publicly recognized, irrespective of whether they are spontaneous or terminated [4]. For example, in Japan a Buddhist ceremony is performed for the *Mizuko* (水子) to facilitate the passing of the soul and comfort the bereaved “parent” [5]. Therefore, for this study, the inclusion criteria for the term “perinatal loss” set out *a priori* encompassed all deaths (spontaneous and terminated) from conception to 28 days post birth.

Estimate from the World Health Organization suggest nine million adolescents over the age of 15 who become pregnant worldwide will experience a perinatal death [6]. Moreover, many adolescents experience gestational death both spontaneously and through intervention globally without seeking medical attention, thus the prevalence is unknown [7,8]. There is an abundance of literature focused on adult perinatal loss responses, particularly in the United States of America, where scholars estimate approximately one fourth of adult pregnancies end in a perinatal death; with African American, older women, and immigrant women experiencing higher rates [9,10]. However, a literature review revealed there is a significant lack of information on perinatal grief during adolescence, and no literature was found that examined the long-term adult impact of an adolescent perinatal loss [11]. The publications identified suggest varying or no academic rigour.

### 1.1 Grief as a response to perinatal death

Grief following perinatal death is a common reaction to the physical severing of an emotional attachment, and the deprivation of future parental dreams where personhood has been ascribed [[12], [13], [14]]. Miscarriage was not recognized in research literature as a grief-inducing event until 1986 [15].

Individual responses to perinatal losses can vary from no impact to intense grief. Hutti (1992) [16] found three factors significantly influence the intensity of grief experienced after a perinatal loss [1]: how real the pregnancy, and fetus or infant, was perceived [2]; how congruent the experience following the loss was compared to the person's perceived ideal regarding how the experience ensued, and [3]; the capacity to respond to events and behave in ways that increased congruence. If grievers are unable to challenge others about behaviours they find unsatisfactory, then they will often feel additional anger and victimization due to the loss experience. Hutti (1992) determined that people who experienced the most heightened grief perceived the pregnancy as real, where the loss experience was viewed as unsatisfactory compared with a desirable experience, and with grievers that felt out of control.

Typically, people who experience intense grief after perinatal loss during adulthood will perceive themselves as “parents” and the death as the loss of an actual child [17]. Conversely, little or no grief was established with people where the pregnancies were not yet attributed with personhood; when the death experience occurred within a supportive environment; and when issues that developed during the event were resolved. Ordinarily, people who experience insignificant levels of grief after perinatal loss will not perceive themselves as “parents” nor will they perceive the perinatal loss as the death of their child [17].

Other adult responses analogous with intense grief following perinatal loss include depression, diabetes, hypertension, increased suicidal ideation and occurrence, anxiety, weight gain, and post‐traumatic stress [[18], [19], [20], [21], [22]]. Additionally, high grief intensity can increase the risk of premature death to nearly two-fold for bereaved parents that may persist up to 15 years beyond the baby's death [23,24]. In addition, studies have found families affected by adult perinatal loss have significantly increased risk of experiencing a significant impact on work productivity and an increased risk of economic deprivation [20,25].

### 1.2 Previous literature

A literature review was conducted prior to commencement of this study, with 20 publications identified that met the inclusion criteria. Nineteen of the 20 publications identified presume, or propound, grief is ubiquitous for adolescents who have experienced a perinatal death. However, no evidence was identified to support this positionality through the implementation of a grief-specific psychometric instrument [17,26]. As empirical research on adult experiences suggests, the perception of personhood is positively correlated with grief responses following perinatal loss. Therefore, the Perinatal Grief Intensity Scale was utilised in this study to ascertain whether adolescent experiences were similarly viewed both at the time of the event and/or across the lifespan [12,13].

### 1.3 The Perinatal Grief Intensity Scale

The Perinatal Grief Intensity Scale was developed as a clinical screening tool, and is used as a standardised instrument to assist healthcare professionals to better predict which people may experience acute grief and the need for continued assessment and/or support following perinatal loss [17,27]. The Perinatal Grief Intensity Scale consists of fourteen items. Hutti (1992) [16] contends that it is not the actual facts surrounding a perinatal loss experience that influence grief intensity. Rather, it is a person's recollected perceptions of the event that influence their subsequent decisions, actions, and resulting grief intensity. The instrument was originally developed based on recollected perceptions of a miscarriage experience [17]. Therefore, it was selected for this study as the most appropriate psychometric instrument to ascertain the recollected perceptions of grief impact both at the time of the adolescent perinatal death and to identify and predict those with continuing elevated scores into adulthood.

## 2 Methods

### 2.1 Instrument: the Perinatal Grief Intensity Scale

The Perinatal Grief Intensity Scale is a 14‐item questionnaire used to evaluate grief intensity after miscarriage, stillbirth, or neonatal death [13]. The PGIS was developed both as a research instrument to assess grief intensity after perinatal death and as a clinical screening tool to help healthcare professionals anticipate who might experience intense grief and require further assessment and support [13,17]. Its construct and refinement was extensively tested within six research studies by the second author to evaluate its validity and reliability. Made publicly available in 2017, the PGIS has excellent face, content, convergent, exploratory, confirmatory, and predictive validity, along with good internal consistency reliability. The optimal cutoff for the PGIS is 3.535, yielding a sensitivity of 61.3 % and a specificity of 84.4 %. The investigators also compared the PGIS to the Perinatal Grief Scale (PGS), which has been the “gold standard” for measuring perinatal grief for over 20 years. The study found that the PGIS is not subordinate to the PGS (AUC = 0.78, 95 % CI 0.68–0.88, p < 0.001) in identifying grief intensity. Moreover, the PGIS uses only 14 items, compared to the 33 items required by the PGS(13).

The PGIS has three subscales: Reality, Congruence, and Confront Others, The instrument has a 4‐point Likert‐type scale, with response options ranging from *strongly agree* to *strongly disagree*. The instrument is used to measure the grief responses of people who have experienced a perinatal loss. The Reality subscale consists of 6‐items that measure the perceived reality of the pregnancy and personhood of the gestation during pregnancy (abortion, miscarriage, and stillbirth) or after birth (neonatal/newborn death). The Congruence subscale consists of 4-items that measure the congruence between the actual loss experience compared with the person's expected ideology regarding how they would like to have experienced the event. The Confront Others subscale consists of 4-items that measure whether someone is able to assert themselves during the perinatal loss experience [13,27]. This instrument was developed through numerous studies of miscarriage, stillbirth and neonatal death, which included studies involving international samples [13,27,28].

### 2.2 Context and setting

A 55-item online survey and semi-structured interviews were used to gather adult women's recollected adolescent miscarriage and abortion experiences from a retrospective bio-psycho-social perspective [29]. This report provides findings associated with 19 of the respondents in six countries who completed the Perinatal Grief Intensity Scale, which was converged within the original 55-item survey. Women were invited to participate in the survey if they met the following selection criteria: were adult females who had experienced a previous miscarriage, stillbirth, neonatal death, or abortion during adolescence, whilst of secondary education age. Anonymised details of the participants are provided in the results section. The survey was available from October 1st, 2019 to December 31st, 2019.

### 2.3 Participant recruitment

Participants were recruited through word of mouth snowballing and social media posts [30,31]. Participants were required to provide informed consent online before access was granted to the study survey. The Perinatal Grief Intensity Scale was embedded within the questionnaire twice: respondents were invited to complete the survey items as they remembered feeling as adolescents at the time of the loss (T1) and asked to complete the items again according to how they currently felt as adults (T2). Respondents were not identifiable, and the anonymous data was downloaded, analysed, and stored in a password protected file. Ethics as process was embedded throughout the study design, implementation, and analyses [[32], [33], [34]].

### 2.4 Data collection

Data were collected in a survey in which the Perinatal Grief Scale (PGIS) was embedded. Participants were also asked if they chose to self-identify as a “bereaved parent”, whether they wanted advice and bereavement support post-loss, and whether such advice and support was received. The participants were instructed to recall how they felt as an adolescent at the time of their loss, and to answer the PGIS as they would have at that time (Time 1). Participants were then instructed to think about how they currently felt as an adult regarding their past perinatal loss, and to answer the T2 PGIS based on these current feelings (T2).

### 2.5 Data analysis

Respondents were not identifiable, and data were downloaded, analysed, and stored in a password protected file. Ethics as process was embedded throughout the study design, implementation, and analyses [[32], [33], [34]].

To derive a Perinatal Grief Intensity Scale score, and to determine frequency results, data were downloaded into and analysed using a properly weighted Excel scoring sheet developed and provided by the second author [17]. The instrument incorporates a total of fourteen questions, situated within three subscales: Confront Others, Reality, and Congruence. The responses were aggregated to provide an overall score of retrospective perceptions of their experience immediately following the loss while they were adolescents (T1) and their perceptions of how they currently feel as adults (T2). Both timelines were measured to ascertain if there were any differences in grief intensity over the participants’ perceived life course. As determined by Hutti et al. (2017) [13], the high grief intensity level was identified as a total score of 3.52 or greater, the mid-range intensity grief group was identified as a total score of ≤3.52 and ≥3.29, and the low-intensity grief group was identified by a total score less than 3.29.

## 3 Results

This is the first study which compares perceptions of grief of women from the same sample to miscarriage versus elective abortion. The completed submissions were received from women in the following countries: England (n = 8), Ireland (n = 3), USA (n = 3), New Zealand (n = 1), Northern Ireland (n = 1), Scotland (n = 1), and two unknown locations. One participant self-identified as black, whilst 18 participants self-identified as white. The participants’ average age was 35 years and the average age when the adolescent perinatal death occurred was 16 years. Forty two percent (n = 8) of the respondents experienced a miscarriage and 58 % (n = 11) experienced an abortion.

### 3.1 Perinatal grief intensity scores as perceived during adolescence (T1)

A summary of participants’ initial retrospective Perinatal Grief Intensity Scores, with the synoptic “Low”, “Medium”, and “High” classifications to specify recollected grief intensity soon after the abortion or miscarriage during adolescence, is presented in Table 1.

**Table 1:** Grief as recollected as an adolescent (T1).

Initial Score as Recollected Following Adolescent Perinatal Death	Totals		Distribution		Percentage	
	Total	Percentage	Miscarriage Total	Abortion Total	Miscarriage Percentage of Total	Abortion Percentage of Total
High	9	47 %	5	4	56 %	44 %
Medium	3	16 %	1	2	33 %	67 %
Low	7	37 %	2	5	28 %	72 %
Totals	19	100 %	8	11		

Table 1 indicates that approximately half (47 %; n = 9) of the 19 participants scored in the high intensity grief category in response to their recollected adolescent perinatal death. Additionally, 16 % (n = 3) scored within the mid-range grief intensity, with the resultant 37 % (n = 7) scoring within the low grief intensity response parameters. Within the adolescent miscarriage group, 56 % (n = 5) scored highly, but high scores were similarly evident within the adolescent abortion group at 44 % (n = 4). The mid-range grief intensity scores were higher in the abortion group at 67 % (n = 4), versus 33 % (n = 1) in the miscarriage group. Low scores were more prevalent in the abortion group (72 %, n = 5) compared to the miscarriage group (28 %; n = 2).

According to the Hutti (1992) perinatal grief intensity theory, women with intense grief would be expected to have higher “Reality” scores when compared with women with low intensity grief (thus, they perceive the fetus as their child and themselves as parents). In addition, women with high grief intensity would be expected to have lower “Congruence” and “Confront others” scores compared with women with low grief intensity.

Data were collected within the survey asking respondents to select whether they self-identified as a “bereaved parent”. Compared with those with low grief intensity, adolescent women with high grief intensity were likely to self-identify as “bereaved parents”, and report higher median T1 scores, higher median T1 “Reality” scores, and lower T1 “Congruence” and T1 “Confront Others” scores consistent with the Hutti (1992) perinatal grief intensity theory. The type of death, that is; miscarriage or abortion, was not a factor in predicting their grief response pattern.

### 3.2 Perinatal grief intensity scores as perceived as adults (T2)

A summary of participants’ Time 2 PGIS scores are presented in Table 2, with the synoptic “Low”, “Medium”, and “High” classifications, to specify perceived grief intensity as adults. We report the two sub-groups identified in this table as the “adult miscarriage group” and the “adult abortion group”.

**Table 2:** Grief as perceived as an adult (T2).

Score as Perceived During Adulthood Following Adolescent Perinatal Death	Totals		Distribution		Percentage	
	Total	Percentage	Miscarriage Total	Abortion Total	Miscarriage Percentage of Total	Abortion Percentage of Total
High	7	37 %	3	4	43 %	57 %
Medium	4	21 %	3	1	75 %	25 %
Low	8	42 %	2	6	25 %	75 %
Totals	19	100 %	8	11		

As reported previously in Table 1, approximately half, 47 % (n = 9) of the 19 participants who completed the Perinatal Grief Intensity Scale scored in the high intensity grief response category when recollecting their early perinatal death experience. As indicated in Table 2, this number was reduced to 37 % (n = 7) when participants reported how they currently feel as adults in response to the perinatal death experienced as an adolescent. Furthermore, the scores in the “low” grief category have increased to almost half at 42 % (n = 8).

The results presented in Table 2 suggest that a normative grief adjustment can occur for females following adolescent perinatal death; with high grief intensity responses reducing to low grief intensity over time.

According to the Hutti (1992) [16] perinatal grief intensity theory, women with intense grief would be expected to have higher “Reality” scores when compared with women with low intensity grief and they would be expected to have lower “Congruence” and “Confront others” scores.

Data collected from the survey illustrated that women who self-identify as “bereaved parents” as adults following the adolescent perinatal death have higher median T2 scores and lower T2 “Congruence” and T2 “Confront Others” scores consistent with the Hutti (1992) perinatal grief intensity theory in adulthood. However, the median T2 “Reality” scores were the same for both groups, indicating adult perceptions of pregnancy loss can change across the life course. Again, the type of death, miscarriage or abortion, was not a factor in predicting the grief response pattern.

### 3.3 Perinatal Grief Intensity Scale score movements

Considering the initial Perinatal Grief Intensity Scale results presented in Table 1, Table 2, as adolescents versus grief intensity results during adulthood, the scores solely illustrate prevailing movements across the scales. Table 3 therefore presents the individual movements within the scales and illustrate that five of the participants showed an increase in scores, not identified when examining the previous tables. Women who experienced adolescent miscarriages were more likely to perceive increased grief intensity over time, while women who experienced adolescent abortions were more likely to perceive similar or lower grief intensity.

**Table 3:** Comparative changes in respondent perinatal grief intensity scale scores over time.

Change in Score over Time	Totals		Distribution		Percentage	
	Total	Percentage	Miscarriage Total	Abortion Total	Miscarriage Percentage of Total	Abortion Percentage of Total
Increased	5	26 %	3	2	60 %	40 %
Same	7	37 %	2	5	29 %	71 %
Decreased	7	37 %	2	5	29 %	71 %
Totals	19	100 %	8	11		

Three of the five women with increased grief intensity scores self-identified as a “bereaved” parent whilst the other two “somewhat disagree” with identification as a bereaved parent. Of the five respondents self-identifying as a bereaved parent in response to the adolescent event, three had experienced a miscarriage and two had experienced an abortion. The five women had living children when submitting their completed questionnaire, which suggests there was no association parental/non-parental status and self-identification as a “bereaved” parent.

## 4 Discussion

Most humans will adapt to a death in their own way in their own time [35,36]. However, cultural dictates, and social expectations can influence the process of adaptation, including pathologising what may be considered normal in antipodal bioecological environments [[37], [38], [39]].

The Perinatal Grief Intensity Scale analyses within this study revealed that despite enduring high-level grief scores for some respondents, there was also evidence of potential post traumatic growth. Post traumatic growth is defined as: “the experience of positive change that occurs as a result of the struggle with highly challenging life crises.” [40].

The results of the Time 1 Perinatal Grief Intensity Scale scores suggest almost half of the respondents exhibiting high grief scores experienced adolescent abortions; this suggests they could be at potential risk for Prolonged Grief Disorder [41,42]. The analyses indicates that: (a) fetal personhood can be ascribed during adolescent pregnancy, regardless of type of death; and, (b) experiencing abortion or miscarriage during adolescence can contribute to high grief intensity.

The dispersal of perinatal grief intensity scores of previous adolescent abortion and miscarriage events across the lifespan (i.e. from T1 to T2, as perceived as an adult) were almost equally distributed within the “high” category; with 57 % (n = 4) of respondents who experienced abortions and 43 % (n = 3) of whom experienced miscarriages. Compared to the scores presented in Table 1 (grief as recollected as an adolescent), Table 2 (grief as perceived as an adult) scores signify a decrement in “high” miscarriage grief intensity over time, however, the abortion grief intensity remained stagnant within the “high” range. The divergence of normative grief adjustment over the lifespan between adolescent abortion and miscarriage experiences may indicate potential for disenfranchised grief or other contributory factors that warrant further investigation [43,44].

The survey analyses also revealed that three of the eight respondents scoring the highest grief intensity scores at Time 2 self-identified as a bereaved parent, one respondent was neutral, and four of the respondents declared that they “somewhat disagree” they were a bereaved parent in response to the adolescent perinatal death. Of the three women self-identifying as bereaved parent, two experienced an adolescent abortion and one experienced an adolescent miscarriage. Of the three women not self-identifying as bereaved parent, two experienced an adolescent abortion and one experienced an adolescent miscarriage. Two participants did not respond to the question asking if they self-identified as a bereaved parent.

The use of the term “parent” is standard lexicon in the literature when referring to females who have experienced a perinatal death, but this study found that varying participant responses to self-identifying as a “parent”, suggests that the universal assumptive use of parent identity is not appropriate. These results also create a paradox; as high PGIS scores may be attributed to the conference of personhood and reality to the potential for life, and scoring highly suggest grief in response to the loss of an attachment. Rejection of the status of “parent” by some high scoring participants in this study raises the question of how women interpret the definition of “bereaved parent”.

Twenty five percent (n = 5) of respondents exhibited increased grief intensity scores over time which may suggest that Adolfsson's (2011) [45] assertion that approximately 10 % of females experience prolonged grief reactions following perinatal loss may potentially be under-representative for adolescent experiences.

For the seven respondents with long-term high grief intensity scores, they all stated that they wanted bereavement support and advice but none was available to them [46]. This indicates a lack of Congruence between the loss they experienced and the loss experience they desired, as well as an inability to ask for (Confront others) the help they needed. According to Hutti et al. (2017, 2018) [13,27] the most intense grief is more likely for women who perceive the baby as real; the loss experience that develops is incongruent with the loss experience that is desired, and the woman feels powerless to do anything about it. Thus, the combined experience of high Reality, low Congruence and low Confront others in these seven women predicted the high grief intensity score they received.

The combined overall results from the initial grief intensity scores, and the subsequent adult post-loss grief intensity scores, indicate that mid-level and intense grief should reduce over time. As with generalisable normative bereavement outcomes, there is an integrative adjustment within the women's lives [37,47,48].

The increase in grief intensity scores over time for some women may indicate a lack of adaptive, normative adjustment and a susceptibility to prolonged grief disorder [13,49]. Of the five respondents with increased grief intensity scores between the immediate adolescent response and adult response, two of the women were located within the US, and three of the women were located in Ireland, thus raising the question of cultural influencers. Further research is needed to elicit cultural normative views and responses to adolescent perinatal deaths [38].

The four respondents who stated their perinatal loss in adolescence was traumatic but had enabled them to change their lives in a significantly positive way were all over 45 years old which may support; “… the finding that coherent positive resolution was predictive of increasing ego‐resiliency from young adulthood to midlife suggests that narrative identity processing may operate as a mechanism of personality change in adulthood … Put simply, changes and new developments in how people interpret their lives may trigger corresponding changes in enduring patterns of thinking, feeling, and behaving—i.e., personality traits—over time” (Pals, 2006, p. 1105) [50].

These findings suggest that normative grief adjustment to adolescent perinatal deaths should not be assumed, i.e. that particular Perinatal Grief Intensity scores lead to particular discrete outcomes. It should be acknowledged that successive events, and environmental contributors, can affect the variability of long term outcomes [51,52].

The Perinatal Grief Intensity Scale was also used to examine adolescent abortion experiences from an adult retrospective perspective to investigate Lee's (2003) [3] contention that long-term emotional costs are largely ignored in Euro-Western societies following these events. The results support the assertion that grief may be omnipresent as a result of an abortion and Euro-Western social apperception of the range of bio-ecological responses to any spontaneous or terminated pregnancy loss, either at the time of the event, or across the lifespan, would be beneficial [4,5].

## 5 Limitations

Firstly, the study constituted a small sample size which necessitates the proviso that further studies would be desirable to support or add further depth to these findings. Whilst the small participant size is a limitation to generalizability, including participants from six countries provides the strength of global perspectives. There is compelling evidence within empirical publications that disseminating research studies comprising of small sample sizes is meaningful. For example, Boddy (2016) declared; “Unique examples of research using a single sample or case but involving new areas or findings that are potentially highly relevant, can be worthy of publication.” (p. 427). Additionally, precedence within the context of child bereavement of small sample sizes for research (n = 26) was evidenced by Tracey (2011) [53] and within the specific area of adolescent perinatal grief (n = 8) by Fenstermacher (2014) [54]. The intended purpose of this study, was to provide a voice to females who endured these events in silence, and to illuminate the complexity of each experience within a bio-ecological framework. Therefore, to preserve this aim, quality, saturation, and depth of findings were prioritised over quantity [[55], [56], [57]].

A second limitation to consider is that the recruitment methods employed invited participants to self-select, so there were potential barriers to entry [58]. These include; (a) the geographic location of the researcher and the physical barriers therein; and (b) the potential for biases implicit within recruitment via the internet [59]. The authors acknowledge an explicit limitation through snowball recruitment: “subjects do not enroll by chance alone and hence the study may be biased and that it may not involve a random sample” (Sadler et al., 2010, 369–374) [60]. Further, data collection for this study straddled the start of the SARS-CoV-2 pandemic lockdowns which may have impacted participant recruitment; whilst physical paper questionnaires and Word document questionnaires were available, none were requested. Moreover, there were potential social barriers due to continued social stigma and political rhetoric, particularly directed at adolescent pregnancy and abortion [[61], [62], [63]].

A third consideration is that his was a retrospective study in which women were asked to recall events that were up to 25 years or more in their past. The retrospective reminiscence of the events being measured may have changed over time due to the fallibility of memory [64,65]. Research suggests that cognitive ageing may reduce the ability to access context specific details from historical events [66]. For older participants, there may be some challenges to the framing and narratives of these early life events. However, Hutti (1992) [16] argues that it is not the actual facts and accurate remembering of a perinatal loss experience that influences grief intensity. Rather, it is a person's recollected perceptions of the event that influence their subsequent decisions, actions, and resulting grief intensity. Furthermore, the Perinatal Grief Intensity Scale scores for this study are retrospectively constructed from an adult perspective; further studies immediately following adolescent perinatal loss utilising the instrument could provide comparison scores for additional insights. Furthermore, due to the retrospective nature of this study, it is difficult to ascertain if the grief scores at Time 2, as detailed in Table 2, apply to the participants as adults, or if the respondents were responding to how they may have felt at any timeline following their perinatal loss experienced during adolescence.

However, the varying geographical locations, and subsequent cultural diversity of this sample is a study strength. This diversity may influence attitudes and responses to adolescent pregnancy and subsequent pregnancy loss. Further in-depth studies should be conducted to ascertain the extent of these differences.

## 6 Conclusions

The application of the Perinatal Grief Intensity Scale in this study has demonstrated the utility of using a psychometric instrument to assist healthcare professionals in identifying adult females who may need support after adolescent perinatal losses and potential impact throughout adulthood [28]. The specific findings for this study also support the construct validity of the Hutti et al. (1998) [17] grief intensity theory. As detailed, within the group of seven women reporting high grief intensity scores at Time 2, all declared that they wanted advice and bereavement support following the loss but that they did not receive any [46].

The implications of this research necessitates the provision of bereavement information, and signposting to medical or psychological support, for anyone who experiences an adolescent miscarriage and abortion.

Further research is needed to determine whether these findings are applicable to other samples of adolescent women who have experienced the later losses of stillbirth or neonatal death, and identification of other variables that may contribute to grief intensity after perinatal loss.

This study has found that for some women, the adverse psycho-social sequelae of adolescent perinatal loss are enduring and grief may increase in response to adult reproductive events. The findings of this study therefore highlight the need for healthcare professionals to screen adult women who have experienced a previous adolescent miscarriage or termination for adverse mental health issues in subsequent pregnancies [28].

## Funding sources

No external funding.

## Data availability statement

All data providing within this article can be accessed on the TARA open access repository at Trinity College Dublin.

URL: http://hdl.handle.net/2262/101593.

## Ethics declaration

Institutional ethics approval was obtained from the School of Education at Trinity College Dublin prior to data collection (ET7259-A-Y-201819).

## CRediT authorship contribution statement

**Caroline A. Lloyd:** Writing – original draft, Project administration, Methodology, Investigation, Formal analysis, Data curation, Conceptualization. **Marianne H. Hutti:** Writing – review & editing, Validation.

## Declaration of competing interest

The authors declare that they have no known competing financial interests or personal relationships that could have appeared to influence the work reported in this paper.
